# Rapid Trust Calibration through Interpretable and Uncertainty-Aware AI

**DOI:** 10.1016/j.patter.2020.100049

**Published:** 2020-07-10

**Authors:** Richard Tomsett, Alun Preece, Dave Braines, Federico Cerutti, Supriyo Chakraborty, Mani Srivastava, Gavin Pearson, Lance Kaplan

**Affiliations:** 1 Emerging Technology, IBM Research Europe, Hursley Park Road, Hursley SO21 2JN, UK; 2 Crime and Security Research Institute, Cardiff University, Friary House, Greyfriars Road, Cardiff CF10 3AE, UK; 3 Dipartimento di Ingegneria dell'Informazione, Università degli Studi di Brescia, Via Branze 38, Brescia 25123, Italy; 4 IBM Research, IBM Thomas J. Watson Research Center, 1101 Kitchawan Road, Yorktown Heights, NY 10598, USA; 5 Networked and Embedded Systems Laboratory, Electrical and Computer Engineering Department, University of California, Los Angeles, 420 Westwood Plaza, Los Angeles, CA 90095-1594, USA; 6 Defence Science and Technology Laboratory, Porton Down, Salisbury, Wiltshire SP4 0JQ, UK; 7 CCDC Army Research Laboratory, 2800 Powder Mill Road, Adelphi, MD 20783, USA

**Keywords:** DSML 1: Concept: Basic principles of a new data science output observed and reported

## Abstract

Artificial intelligence (AI) systems hold great promise as decision-support tools, but we must be able to identify and understand their inevitable mistakes if they are to fulfill this potential. This is particularly true in domains where the decisions are high-stakes, such as law, medicine, and the military. In this Perspective, we describe the particular challenges for AI decision support posed in military coalition operations. These include having to deal with limited, low-quality data, which inevitably compromises AI performance. We suggest that these problems can be mitigated by taking steps that allow rapid trust calibration so that decision makers understand the AI system's limitations and likely failures and can calibrate their trust in its outputs appropriately. We propose that AI services can achieve this by being both interpretable and uncertainty-aware. Creating such AI systems poses various technical and human factors challenges. We review these challenges and recommend directions for future research.

## The Bigger Picture

This article is about artificial intelligence (AI) used to inform high-stakes decisions, such as those arising in legal, healthcare, or military contexts. Users must have an understanding of the capabilities and limitations of an AI system when making high-stakes decisions. Usually this requires the user to interact with the system and learn over time how it behaves in different circumstances. We propose that long-term interaction would not be necessary for an AI system with the properties of interpretability and uncertainty awareness. Interpretability makes clear what the system “knows” while uncertainty awareness reveals what the system does not “know.” This allows the user to rapidly calibrate their trust in the system's outputs, spotting flaws in its reasoning or seeing when it is unsure. We illustrate these concepts in the context of a military coalition operation, where decision makers may be using AI systems with which they are unfamiliar and which are operating in rapidly changing environments. We review current research in these areas, considering both technical and human factors challenges, and propose a framework for future work based on Lasswell's communication model.

## Teaser

We introduce the concept of rapid trust calibration for AI decision support, and propose how this can be achieved by building AI systems that are both interpretable and uncertainty-aware. We provide a literature review of these research areas and describe a military scenario illustrating the relevant concepts. We propose a framework inspired by Lasswell's communication model to structure future work in this area.

## Main Text

### Introduction

The promise of artificial intelligence (AI) systems to analyze and rapidly extract insights from large amounts of data have stimulated interest in applying AI to problems in complex domains involving high-stakes decision making.^1^, ^2^, ^3^ In such domains, human experts are relied upon to form a final decision supported by the outputs of the AI, forming a human-AI team. Several studies have shown that the performance of such teams can be greater than the performance of the human or the AI alone,^4^^,^^5^ suggesting that each member of the team is able to compensate for the other's weaknesses. For this to happen, the human must build an adequate mental model of the AI and its capabilities. Failing to build a suitable mental model will result in the human miscalibrating their level of trust in the AI, and the human-AI team will perform poorly.

In this Perspective, we argue that AI systems can help human team-mates build suitable mental models by giving explanations of how their outputs were arrived at (providing interpretability) and estimates of the uncertainty in their outputs. These two factors help the human to understand both what the AI “knows” and what the AI does not “know.” These requirements are motivated by the scenario of AI-supported decision making in future military coalition operations.^6^ Here, we describe the coalition setting and how AI systems may be deployed in this setting to support human decision making. We use this to motivate our proposed requirements of interpretability and uncertainty awareness for robust AI-supported decision making. We discuss the technical challenges and human factors challenges posed by these requirements, and highlight promising recent work toward solving these problems.

### AI in Coalition Operations

The context of our AI research is the Distributed Analytics and Information Science International Technology Alliance (DAIS-ITA) (https://dais-ita.org/), which takes future military coalition operations as the motivating setting. Coalitions may be formed quickly to respond to rapidly changing threats, and operations will be conducted jointly across five domains (land, sea, air, space, and cyber),^7^ presenting a complex and highly dynamic environment for military decision makers to understand. To help make sense of the ongoing situation in a coalition operation, militaries will increasingly rely on AI technologies to obtain insights that can assist human decision makers.^8^, ^9^, ^10^

The envisaged scenario poses several challenges for current AI techniques.^11^

1 Although large amounts of data may be collected during rapidly evolving operations, there will not be enough time or resources to clean and label all of these data for (re)training models.
2 During the course of an operation the situation may change dramatically, meaning that data will not be generated from a static distribution but will drift over time.
3 Adversaries may attempt to manipulate data to confuse the coalition's AI systems and, thereby, the decision makers.
4 Due to the operational environment the network supporting the coalition may be slow and unreliable, meaning that access to large, central computing power is not guaranteed. AI services will therefore be distributed over low-power devices at the edge of the network, communicating peer-to-peer. The set of services available to an analyst at any given time will change based on their physical location, the network state, and dynamic prioritization of tasks across the network.

The first three points are about the nature of the data: only small amounts of data will be available for retraining during the course of the operation, and these data may be unreliable. The AI services will therefore be operating on out-of-distribution data, where guarantees cannot be made about their performance. The final point means that human analysts will be interacting with a variety of AI services with which they may be unfamiliar. The rapid formation and dynamic nature of the coalition operation may not allow humans to build up experience of the specific AI services through training prior to, or repeated use during, the operation. These four factors will adversely affect the overall performance of the human-AI team without mitigations to improve trust calibration.

In the next section we describe the concept of trust calibration, how this affects human-AI team performance, and how it could be improved by developing interpretable and uncertainty-aware AI systems. We provide definitions of these and related terms (including our usage of “AI”) in Table 1.

**Table 1:** Glossary of Terms, Defined in Relation to Human-AI Teams

AI	artificial intelligence: the property of a computer or machine to display “intelligent” behavior more usually associated with humans or non-human animals, and the methods and technologies used to achieve this. In this article we focus largely on AI using machine learning to support human decision making
AI service	a stand-alone piece of software implementing a single AI functionality, e.g., IBM Watson Visual Recognition (https://cloud.ibm.com/catalog/services/visual-recognition, accessed April 28, 2020)
AI system	a system composed of one or more AI services. Each service in the system may be owned or operated by a different organization or coalition partner. Where unambiguous, we refer simply to “an AI” to mean an AI system
Trust level	the extent to which the human believes the AI's outputs are correct/useful for achieving their current goals in the current situation. While trust is a very broad and nuanced topic,^12^, ^13^, ^14^ we restrict ourselves to this narrower definition to help focus our discussion
Trustworthiness	the degree to which the AI warrants trust from the human
Trust calibration	the process through which the human sets their trust level appropriately to the AI's trustworthiness
Interpretable	a property of the AI system that allows a human to understand the reasons for the system's output
Explanation	information provided by the AI system to the human that provides reasoning around why the system produced a specific output
Aleatoric uncertainty	uncertainty caused by inherent unpredictability in the system (e.g., the outcome of a coin toss or dice roll)
Epistemic uncertainty	uncertainty caused by a lack of knowledge, reducible by observing more data

Adapted from Hüllermeier and Waegeman,^15^ Lee and See,^16^ Nilsson,^17^ and Tomsett et al.^18^

### Results

#### Rapid Trust Calibration for Robust Human-AI Team Decision Making

To obtain the greatest benefit from using decision-support AI, the human must have an appropriately calibrated level of trust in the system.^16^^,^^19^ Trust is well calibrated when the human sets their trust level appropriately to the AI's capabilities, accepting the output of a competent system but employing other resources or their own expertise to compensate for AI errors; conversely, poorly calibrated trust reduces team performance because the human trusts erroneous AI outputs or does not accept correct ones.^16^^,^^20^ Bansal et al.^21^ formalize this by measuring how well humans learn and respond to the AI's error boundary (the boundary separating inputs that are correctly classified versus those that cause the AI to make mistakes). However, AI systems dealing with high-dimensional data and/or many classes will have error boundaries that are hardly self-explanatory. In the coalition setting, the human may not have the opportunity to learn the error boundary: the AI services they use may differ from those they have been trained to use (e.g., if they belong to other coalition partners), and operate on data that differs from the training data, resulting in unpredictable error boundaries. When every decision is high-stakes, the human must be able to calibrate their trust in the AI quickly and adjust their trust level on a case-by-case basis. We refer to this process as rapid trust calibration.

Rapid trust calibration can be posed as a problem of communication: the AI system must quickly communicate its abilities and limitations to the user. We therefore follow van der Bles et al.^22^ in suggesting turning to Lasswell's^23^ model of communication to inform what facets of AI to human communication may affect trust calibration, and therefore where to focus research efforts. Lasswell's model asks us to identify the following: who says what in what form to whom with what effect. Braddock^24^ proposed also considering the circumstances and the purpose of the communication. We include circumstances, as these will vary greatly even within the coalition context, and purpose, as it helps make explicit the goals of the communication. In the context of AI-supported decision making, the “who” in question is the AI system, the “to whom” is the human decision maker, and the “purpose” of the communication is to improve the human's decisions. The “effect” of the communication will depend on what is communicated, in what form, and under what circumstances, as well as the characteristics of the decision maker to whom it was transmitted. Structuring future research using this model will help both in narrowing down research questions and in identifying the research's applicability to different settings.

We propose that for rapid trust calibration, what is communicated should include explanations for the AI's outputs (providing interpretability) and the AI's level of uncertainty. This suggestion is informed by the decision-making literature, which suggests that trust calibration requires understanding a system's capabilities (provided through interpretability), and the reliability of the system's outputs (provided through uncertainty estimates).^19^ In the next sections we further justify this view, and provide a concrete example of how these two facets could enable rapid trust calibration in a coalition operation. We turn to the associated technical challenges in the Discussion section, as well as considering the effects of the form and circumstances of the communication and the characteristics of whom is being communicated with.

##### Why Interpretability?

Doshi-Velez and Kim^25^ argue that interpretability is necessary when the AI and human agents have mismatched objectives. This is likely in practice, especially in complex decision scenarios: AI systems are trained to optimize a narrow set of objectives that can be conveyed mathematically, but their outputs are then used by the human to inform a decision that was never expressed in these objectives. Consider a vision model that has been trained to recognize different kinds of vehicles in images. This model may be used by an analyst to assess the threat level of an enemy force. The downstream decision informed by the model really needs to consider the capabilities of, and threats posed by, these vehicles; the specific category of the vehicles themselves is not directly relevant. However, the AI has no concept of vehicle capabilities: it has been trained to recognize them based only on image data. Vehicles with different capabilities may have similar visual features in the training data and thus be more frequently confused by the model. In this situation, appropriate explanations could help reveal this problem to the human by highlighting the relevant visual features, revealing the mismatch between the AI's interpretation of the image and the human's and allowing them to update their mental model of the AI's capabilities.^26^

The training data itself, in addition to the mechanics of training, also contribute to the objective mismatch problem. We generally assume the training data to be adequately representative of the distribution we are trying to learn. For many problems and many kinds of data, this assumption does not hold. In the coalition setting, models may be trained on data gathered during previous operations, which are not adequately representative of the new scenario to which they are being applied. The data may be flawed in any number of unknown ways,^27^ leading to unquantified biases in the models that are difficult to identify prior to deployment. Suitable explanations that identified these biases during operation would improve the human's mental model of the AI's abilities.

##### Why Uncertainty?

Interpretability gives the human access to what the AI system has learned, and how it uses that knowledge in producing outputs. Understanding what the AI does not know is also extremely important for creating a suitable mental model of the AI's capabilities.^21^^,^^28^^,^^29^ To do this, the AI system must be able to estimate the uncertainty in its outputs. Uncertainty is often described as a single concept, although several authors have made attempts to categorize different kinds of uncertainty.^30^^,^^31^ Weisberg^32^ divides uncertainty into components of doubt and ambiguity; doubt may be quantified as a probability while ambiguity results from a lack of knowledge. Doubt and ambiguity roughly correspond to a distinction commonly made in the machine learning and statistics literature between aleatoric and epistemic uncertainty. Aleatoric uncertainty (doubt) represents uncertainty inherent in the system being modeled (e.g., through stochastic behavior) while epistemic uncertainty (ambiguity) is the uncertainty due to limited data or knowledge.^15^^,^^33^^,^^34^ For example, an uncertainty-aware image classifier should exhibit high aleatoric uncertainty for images that are similar to those it was trained on, but that do not contain adequate distinguishing features for choosing between classes; it should estimate high epistemic uncertainty for images that look different from those in the training set (e.g., a noisy image, or an image of an unknown class of object). Aleatoric uncertainty is irreducible while epistemic uncertainty can be reduced by observing more data. Humans seem to think and talk about these kinds of uncertainty differently—using words like “sure” and “confident” to refer to epistemic, and “chance” or “probability” to refer to aleatoric uncertainty^35^—even if only subconsciously and despite their frequent conflation in mathematical modeling.^22^^,^^36^

It is particularly important to understand epistemic uncertainty in the coalition scenario.^37^ At the start of an operation, coalition partners will deploy AI systems trained on historical data. This is unlikely to adequately capture the data distributions present in a new setting because of differences in the environment and changes in adversaries' behaviors. Much of the actual input data to the AI during the coalition operation will therefore be out of distribution (not part of the distribution the AI was trained on), which will cause errors no matter how many data the system was trained on previously.^38^ As an operation continues, models may be retrained on more relevant data, but the amount of data available will be limited (and possibly conflicting and of low quality). As the AI's knowledge will always be constrained by these factors, communicating its epistemic uncertainty is crucial for ensuring that the human is able to build a mental model of what the AI does not know.

#### Example Scenario

**Figure 1:**
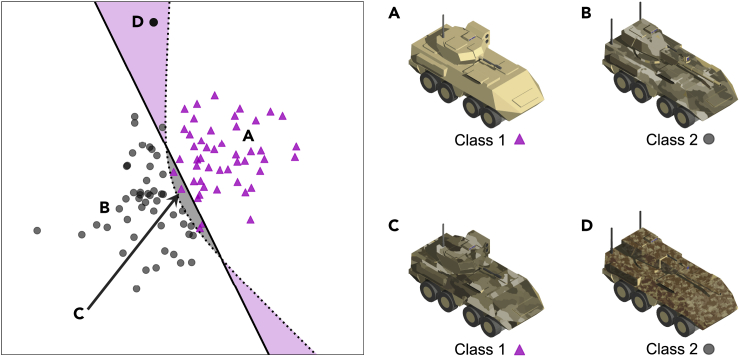
Example Scenario (A and B) A coalition-operated AI service (an image classifier) has been trained to distinguish between different kinds of enemy vehicle. The plot on the left shows a 2D projection of the latent feature space of the classifier, with inputs from two different classes of vehicle depicted as magenta triangles (class 1) and black circles (class 2). Example inputs for these two classes are shown on the right of the figure (A and B). The human (ground truth) decision boundary is the dotted black line, and the classifier's learned decision boundary is the solid black line: regions where the classifier will make errors are shaded (gray for class 1 inputs mistaken for class 2, magenta for class 2 inputs mistaken for class 1). A and B are far away from the decision boundary but well within the learned data distribution, so should be classified with low epistemic uncertainty. (C) An input that confuses the classifier, because it has learned to rely on camouflage as a feature to distinguish between vehicle types. (D) An input that is far from the learned distribution, because vehicles with this camouflage pattern were not in the training data: it should be classified with high epistemic uncertainty.

The following scenario, illustrated in Figure 1, demonstrates how both interpretability and uncertainty communication could improve human-AI team performance. Consider an analyst assessing the level of enemy activity over the area of operations who has access to various autonomous sensors and AI services deployed by the coalition in forward positions, including a camera feeding a neural network model that can identify different kinds of enemy vehicle. During their surveillance task, a vehicle is spotted and classified by the model. On examining the explanation for the classification, the analyst sees that the model has focused on the vehicle's camouflage pattern. As the analyst knows that the enemy uses several camouflage patterns and that these are not vehicle dependent (this might not have been known when the model was originally trained), they infer that the model may be mistaken in this case (see Figure 1C). They have therefore been able to calibrate their trust appropriately and have updated their mental model of the AI's capabilities.

**Figure 2:**
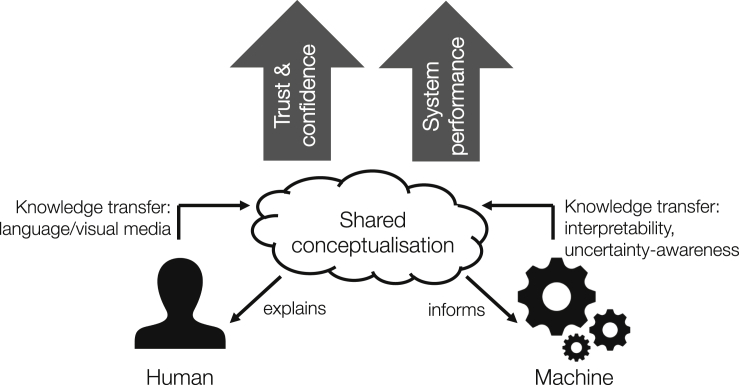
Human-Agent Knowledge Fusion for Improved Confidence and Performance in Support of Better Decision Making Adapted from Preece et al.^11^

During the same surveillance operation, another vehicle is classified by the model with high epistemic uncertainty (Figure 1D). Unknown to the analyst, the enemy has developed a new camouflage pattern and has started deploying these vehicles in the area of operations. As this pattern has not appeared in the model's training data, it reports high epistemic uncertainty, thus alerting the analyst that they should not trust its classification output. In this case, providing only an explanation could be misleading: the input image is out of distribution, so the region of latent space it is mapped to is not meaningful, potentially resulting in confusing or meaningless explanations.

Although this example is somewhat contrived and overly simplified, it helps illustrate how interpretability and uncertainty awareness contribute toward rapid trust calibration. We can also transfer this simplified scenario more easily to other domains. In medical imaging diagnostics, for example, appropriate interpretability would allow a radiologist to assess how well the AI system has aligned with their own expert knowledge, enabling them to identify the model's biases for each new case. Epistemic uncertainty would allow them to quickly identify gaps in the AI's training—inevitable when models are deployed at different locations with diverse patient populations.

### Discussion

#### Technical Challenges: Who Communicates What

Before interpretability and uncertainty estimates can be used to improve human-AI decision making, we need reliable methods for creating both. This poses difficult technical challenges that have yet to be fully solved.

##### Interpretability

One solution is to use models that are intrinsically interpretable so that accurate explanations can be produced naturally from the model structure. Some authors have suggested that this approach is the only acceptable solution for high-stakes decision making due to both technical and conceptual limitations in trying to create explanations for uninterpretable models.^39^ Indeed, much current research into producing “post hoc” explanations^40^ of (uninterpretable) neural network outputs has resulted in techniques that are difficult to validate,^41^ with some failing basic sanity checks.^42^ This would preclude the use of neural network models for high-stakes decision support.

However, their ability to automatically learn features from low-level data means that neural networks perform well on domains for which features are difficult to engineer by hand, e.g., learning from images, audio, video, sensor streams, and natural language. These are exactly the kinds of data sources we are interested in using during coalition operations, as well as other high-stakes domains such as medicine and autonomous driving. Combining neural networks' powerful representational capacity with techniques that improve their inherent interpretability is an active research area, with a variety of approaches showing promise.^43^, ^44^, ^45^

##### Uncertainty Quantification

Quantifying epistemic uncertainty requires the model to have a means of accurately estimating how far away new inputs are from the data distribution it was trained on. A common approach is to use Bayesian methods, whereby epistemic uncertainty is captured as uncertainty in the model parameters^33^ or as uncertainty in function space using, for example, Gaussian processes.^46^ Another promising approach is that of evidential learning,^47^^,^^48^ whereby inputs are mapped to the parameters of a Dirichlet distribution over classes. Smaller parameter values represent less evidence for a class, producing a broader distribution representing greater epistemic uncertainty. This approach also benefits from a direct mapping to the framework of subjective logic.^49^ Subjective logic has many appealing properties for AI applications in the coalition setting, allowing aleatoric and epistemic uncertainty to be considered during logical reasoning operations as well as providing a framework for incorporating subjective evidence from sources with different levels of trust.^50^

These methods all have associated problems that require further research for them to be overcome. Bayesian methods rely on sampling approaches that increase their computational cost at inference time while Gaussian processes present issues when scaling to high-dimensional problems.^51^ The uncertainty estimates are dependent both on the specifics of the approximations and on the prior probability distributions used. The evidential learning approach learns a generative model to create out-of-distribution samples so that the classifier can be explicitly taught the input regions it should be uncertain about,^48^ but this introduces complications in the training process. The evaluation of epistemic uncertainty estimates is also challenging: they are fundamentally subjective^22^ with cases of high epistemic uncertainty being largely driven by the prior, so that defining metrics to assess the validity of these estimates is conceptually difficult.

##### Explanations of Uncertainty, and Uncertainty in Explanations

Creating explanations for the causes of model uncertainty, and estimating the uncertainty in explanations of outputs, are relatively underexplored areas. Epistemic uncertainty could arise because an input is unlike the training data in any feature or because it contains a set of known features in a previously unseen combination. Distinguishing between these cases may be helpful for the decision maker, potentially pointing toward different lines of further inquiry. These kinds of explanations have only recently begun to be explored.^52^, ^53^, ^54^

Explanations may also have some uncertainty attached to them, especially if they summarize the model's reasoning trace. As far as we are aware, only one study has investigated uncertainty in explanations: Merrick and Taly^55^ calculated the variance of Shapley values, which are a commonly used method to estimate feature importance.^56^ This is also an underexplored research area, yet one that could have important implications for assessing explanation reliability.

#### Human Factors Challenges: What Form, What Circumstances, to Whom

However good the technical solutions for interpretability and uncertainty awareness become, they will be useless unless they can be made accessible and useful to humans. AI and data science researchers must engage and collaborate with human computer interaction (HCI), psychology, and social science researchers to find the best approaches for facilitating rapid trust calibration.

##### Automation Bias and Algorithm Aversion

Automation bias is a well-studied phenomenon that hinders trust calibration.^57^^,^^58^ It occurs when humans accept computer outputs in place of their own thinking and judgment, leading them to place too much trust in algorithmic outputs. Various studies have looked at different factors affecting automation bias, including the cognitive load of the user,^58^ the accountability of the user in the decision process,^59^^,^^60^ and their level of expertise and training.^61^ Conversely, algorithm aversion occurs when humans disregard algorithms that actually perform better than humans, thus affecting trust calibration in the opposite direction to automation bias.^62^ This effect has been studied most in the context of forecasting tasks, whereby humans tend to lose trust in an algorithm's advice very rapidly in response to errors;^63^ by contrast, trust in other humans who make the same errors reduces more slowly.^64^ Other experiments have produced conflicting results, suggesting that only expert forecasters are susceptible to algorithm aversion while lay users are more likely to trust algorithmic advice.^65^

The possible influences of automation bias or algorithm aversion on AI used for decision support are unclear. Some results regarding the tendency of explanations to cause humans to be overly trusting of conventional decision aids seem to transfer to AI-based aids,^66^^,^^67^ although the effects will be dependent on the particular characteristics of the explanations provided.^68^ There are many different kinds of explanation that an AI system could supply,^69^, ^70^, ^71^ so future research on the impact of different kinds of explanation on trust calibration should be guided by knowledge gained in the social sciences on how humans understand explanations.^72^^,^^73^ Providing uncertainty estimates along with explanations may also improve trust calibration, but research remains to be done in this area. In particular, humans are not naturally competent at reasoning with probabilities, as described in the next section.

##### Communication of Uncertainty

Van der Bles et al.^22^ surveyed epistemic uncertainty communication about facts, numbers, and science, but found no systematic studies of how epistemic uncertainty affects decision making (noting that many studies do not distinguish epistemic from aleatoric uncertainty). However, many papers have looked at how humans understand probabilistic information, including most famously those by Kahneman and Tversky.^74^, ^75^, ^76^ This work demonstrated that humans are not good at reasoning with probabilities, regularly committing errors such as the base-rate fallacy.^77^ Research since has suggested that some such errors can be mitigated by presenting probabilities in a form closer to humans' natural mental representations of them as frequencies of events.^78^ Combined with the observation that people naturally describe aleatoric and epistemic uncertainties differently,^35^ this suggests that finding suitable forms to present probabilistic uncertainty information to users could allow them to use this information to improve their trust calibration in an AI system. Some studies have found that particular non-probabilistic representations of uncertainty or confidence can lead to improved trust calibration in specific settings,^79^^,^^80^ but further work is needed to understand the best way to represent different kinds of uncertainty under different circumstances and how best to combine the characteristics of interpretability and uncertainty awareness.

#### Suggestions for Researchers and Practitioners

The discussion above leads us to the following suggestions for future research into these topics, as well as recommendations for data science practitioners working with decision-support AI today.

##### Researchers

Interpretability and uncertainty awareness are currently very active topics in AI research, particularly in the deep-learning community where standard methods provide neither of these properties.^81^, ^82^, ^83^, ^84^, ^85^ This research still lacks a deeper appreciation of how humans, with various levels of background knowledge and differing roles and goals, interpret different explanations and uncertainty information. Although important studies from the HCI community have probed these questions,^67^^,^^86^^,^^87^ more collaborative work between AI and HCI researchers, as well as statisticians and others experienced in communicating about uncertainty, will be crucial for focusing technical research toward developing methods that are actually useful for different human stakeholders.^88^ We suggest that researchers from these fields use Lasswell's communication model^23^^,^^24^ outlined above as a common reference to help frame their discussions.

##### Data Science Practitioners

Although further research is necessary to establish best practices for building interpretable, uncertainty-aware AI systems, data scientists and developers can start incorporating these ideas into the AI decision-support systems they build. Explanation is important, but the provision of explanatory mechanisms in AI systems needs to be driven by clear requirements (in software engineering terms) specific to the various classes of user/stakeholder.^18^ We suggest that developers focus their efforts on enabling rapid trust calibration by framing user requirements in terms of (1) explanations for the AI's outputs (for interpretability) and (2) communication of the AI's level of aleatoric and epistemic uncertainty, and ensuring close collaboration with all relevant stakeholders to ensure appropriate communication of these factors. Again, Lasswell's communication model^23^^,^^24^ may prove helpful for framing these collaborations.

### Conclusion

AI holds great promise for use in decision support. To fulfill its potential, we must create AI systems that help humans to understand their strengths and weaknesses, allowing rapid trust calibration. This is particularly important in military operations, where AI services are likely to encounter out-of-distribution data, and operators will not have time to build up adequate mental models of the AI's capabilities through training or interaction. In this Perspective, we have proposed building AI services that are both interpretable and uncertainty-aware, illustrating how these two features together could facilitate rapid trust calibration. We suggest using the framework provided by Lasswell's communication model to structure future research efforts.

Although we have focused on one-way communication from AI to human, our long-term goal is to enable bidirectional communication so that the human-AI team can form a shared conceptualization of the problem space they are tackling (see Figure 2). This approach has been studied in classical (“good old-fashioned”) AI, leading to the creation of ontology technologies culminating in the Semantic Web;^89^ our prior work in this area focused on controlled natural language as a medium for human-machine collaboration, allowing natural and artificial agents to operate on the same linguistically expressed information.^90^ The recent breakthroughs in AI, founded on subsymbolic models, are compatible with these approaches only if the AI's internal representations can be externalized in communicable terms, and those same terms can be used by the human to inform the AI's internal representations. This creates a system that is both explainable and tellable: we can provide it with new knowledge directly in human-understandable terms. This not only has the potential to benefit the human team-member's trust calibration^91^ but also allows the AI to assess its team-mate's knowledge and biases, and thus calibrate its trust in the human, potentially allowing it to alter its communication strategy to account for the human's flaws. To create tellable systems, we see promise in approaches that combine elements of symbolic AI with successful subsymbolic approaches to allow humans and machines to operate on shared conceptualizations of the world.^92^^,^^93^ How this can best be achieved is currently a key open problem in AI.^94^
